# Draft Genome Sequence of Bacillus subtilis Strain FB6-3, Isolated from Fermented Bamboo Shoot

**DOI:** 10.1128/MRA.01319-18

**Published:** 2018-11-15

**Authors:** Olivia Khunjan, Piyush Pandey

**Affiliations:** a Department of Microbiology, Assam University, Silchar, Assam, India; Indiana University Bloomington

## Abstract

Here, we report the draft genome sequence of Bacillus subtilis strain FB6-3, isolated from fermented bamboo shoot samples (*Soibum*) from Manipur, India. The genome was constructed to facilitate studies in evolution of the genetic code. The *de novo* assembly resulted in a chromosome size of 4,192,717 bp and 3,885 coding sequences.

## Abstract (precis)

Here, we report the draft genome sequence of Bacillus subtilis strain FB6-3, isolated from fermented bamboo shoot samples (*Soibum*) from Manipur, India. The genome was constructed to facilitate studies in evolution of the genetic code.

## ANNOUNCEMENT

Bacillus subtilis is a prominent species of aerobic endospore-forming bacteria (1), which also includes differences with respect to nutritional requirements, growth conditions, and DNA base composition (2). B. subtilis FB6-3 was isolated from fermented bamboo shoot samples, which are an ethnic food of Manipur. In fact, the isolate was found to have excellent fermentative abilities, and we therefore experimented and optimized for production of fermented bamboo shoots by a scientific method, in place of the traditional method, which will be reported elsewhere. The FB6-3 strain was isolated along with several other culturable bacteria from fermented bamboo shoots. For this, 1.0 g of fermented bamboo shoot sample was crushed in sterile water, and suitable dilutions were plated on nutrient agar medium and incubated at 30°C for 48 h (3). Pure culture of FB6-3 was maintained on nutrient agar at 4°C. Genomic DNA was isolated from the bacterial culture using a cetyltrimethylammonium bromide (CTAB) and the phenol-chloroform extraction method, followed by RNase A treatment and purification. The isolated DNA was quantified using NanoDrop by determining *A*_260_/*A*_280_ ratio. The isolate was found to be closely related to B. subtilis on the basis of 16S rRNA gene sequence similarity. To determine the genome sequence of B. subtilis FB6-3, whole-genome shotgun sequencing was performed using paired-end sequencing libraries and a TruSeq Nano DNA library prep kit (Illumina). The DNA was fragmented by using a Covaris M220 ultrasonicator, which generates double-stranded DNA (dsDNA) fragments with 3′or 5′ overhangs. The fragments were then subjected to end repair, followed by adapter ligation to the fragments. The products were then PCR ampliﬁed with the index primer, as described in the kit protocol, and sequenced using a NextSeq 500 instrument.

The average DNA library fragment size was found to be 487 bp. The paired-end data were assembled to obtain high-quality clean reads, using Trimmomatic v0.35 to remove ambiguous reads, adapter sequences, and low-quality sequences. These reads were trimmed with a quality score threshold of 20 and a length cutoff 20 bp. *De novo* assembly of the sample was accomplished using SPAdes v3.11.1 (4). The procedure for genome annotation was done by using the Rapid Annotations using Subsystems Technology (RAST) server (5). The average GC content of contigs was approximately 44.1%. The contigs were assembled into a genome by reference-based assembly via CONTIGuator (6), using B. subtilis subsp. subtilis strain NCD-2 (GenBank accession number CP023755) as a reference. The total length of the genome was 4,192,717 bp, while the contig *N*_50_ was 74,814. A total of 4,105 genes were predicted in B. subtilis strain FB6-3, including 3,885 open reading frames (coding sequences [CDS]), along with 83 tRNAs.

### Data availability.

This whole-genome shotgun project has been deposited in GenBank under BioProject accession number PRJNA480558, with corresponding GenBank accession number CP032089 and SRA accession number SRR8083351.
